# Recent Advances in the Green Synthesis of Active *N*-Heterocycles and Their Biological Activities

**DOI:** 10.3390/ph16060873

**Published:** 2023-06-13

**Authors:** Suman Majee, Shilpa, Mansi Sarav, Bimal Krishna Banik, Devalina Ray

**Affiliations:** 1 Amity Institute of Click Chemistry Research and Studies, Amity University, Sector 125, Noida 201313, Uttar Pradesh, India; smajee@amity.edu; 2 Amity Institute of Biotechnology, Amity University, Sector 125, Noida 201313, Uttar Pradesh, India; 3 Department of Mathematics and Natural Sciences, College of Sciences and Human Studies, Prince Mohammad Bin Fahd University, Al Khobar 31952, Saudi Arabia

**Keywords:** *N*-heterocyclic compounds, green synthesis, drug design and development, bioactivity, pharmaceutical activity

## Abstract

*N*-heterocyclic scaffolds represent a privileged architecture in the process of drug design and development. It has widespread occurrence in synthetic and natural products, either those that are established or progressing as potent drug candidates. Additionally, numerous novel *N*-heterocyclic analogues with remarkable physiological significance and extended pharmaceutical applications are escalating progressively. Hence, the classical synthetic protocols need to be improvised according to modern requirements for efficient and eco-friendly approaches. Numerous methodologies and technologies emerged to address the green and sustainable production of various pharmaceutically and medicinally important *N*-heterocyclic compounds in last few years. In this context, the current review unveils greener alternatives for direct access to categorically differentiated *N*-heterocyclic derivatives and its application in the establishment of biologically active potent molecules for drug design. The green and sustainable methods accentuated in this review includes microwave-assisted reactions, solvent-free approaches, heterogeneous catalysis, ultrasound reactions, and biocatalysis.

## 1. Introduction

**Figure 1:**
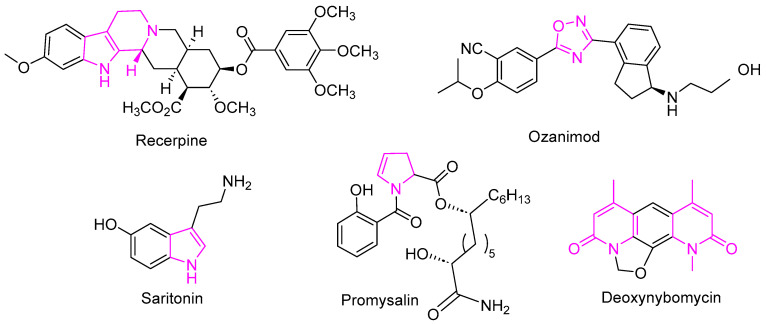
Natural-product-derived *N*-heterocyclic drugs and bioactive compounds.

**Figure 2:**
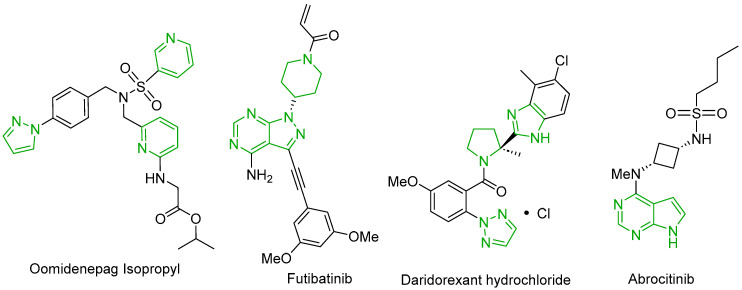
Synthetic *N*-heterocyclic drugs and bioactive compounds approved by FDA recently.

*N*-heterocyclic compounds have become progressively in demand for their exclusive structural identity exhibiting myriad medicinal and pharmaceutical activities [1,2,3,4,5,6,7,8,9,10,11,12,13,14,15,16]. The saturated and unsaturated *N*-heterocyclic analogues have exceptionally interesting architecture for drug design and development [7,17,18,19,20,21]. Numerous naturally occurring *N*-heterocyclic scaffolds are the major components of several pharmaceuticals, antibiotics, nucleic acids, etc., which unveil their importance and compulsion in drug discovery [22,23]. The majority of the FDA-approved drugs reflect the unique significance of *N*-heterocyclic scaffolds in realizing the true essence of drug discovery [7,21,24]. The heterocyclic counterpart is capable of exhibiting weak coordination ranging from hydrogen bond to п-stacking interactions in addition to prominent electronic effects [23]. The enhanced affinity to selective binding of these heterocyclic entities with targeted receptors and enzymes can be connected to their modulated solubility. The existence of *N*-heterocycles as primary units in various amino acids, purine, and pyrimidine bases, which are the essential components of DNA and RNA, divulges the irreplaceable identity of these moiety in nature and unnatural sources. The unique ability of nitrogen in heterocycles for diverse binding with the biological targets enriches the process of drug discovery [25,26,27,28,29,30,31]. Various natural-product-derived N-heterocyclic FDA-approved drugs are available on the market. Some are under phase III clinical trial whereas several others have shown promising bioactivity to take is forward in the process of drug discovery [32,33,34]. A few such natural-product-derived *N*-heterocyclic compounds are displayed in Figure 1, whereas the synthetically prepared drug molecules are represented in Figure 2. The structural variations in the substituents attached to the core *N*-heterocyclic scaffold also contribute to improved bioactivity, inspiring the researchers to focus on exclusive synthetic strategies for their simple and efficient access [35,36,37,38].

There are innumerable synthetic protocols in the development of *N*-heterocyclic analogues [39,40,41,42]. However, the synthetic methods focusing on the greener version are comparatively fewer, leaving ample scope for exploring this area of research [43]. Among them, the environmentally benign procedures leading to convenient access to biologically active complex *N*-heterocyclic frameworks with medicinal and pharmaceutical significance are rarer [19,44]. The pharmaceutical, agrochemical, and many other industries are regularly generating tremendous hazardous and toxic wastes, elevating environmental burden significantly. Hence, the urgent requirement of efficient synthetic protocols that complement the current guidelines for safety and sustainability is of high priority. Therefore, efforts are devoted towards the development of eco-friendly synthetic strategies that rely on the realm of green chemistry principles. The most pertinent future approaches to accomplish this objective are distinctly represented in this review, i.e., employing various green and sustainable protocols involving multicomponent reactions (MCR), microwave chemistry, green/renewable solvents, heterogeneous catalysis, and solvent-free conditions. Additionally, the waste generation in a reaction should be minimized, possibly with fewer purification requirements owing to the features of green and sustainable approach. Presently, the green and sustainable strategy for the synthesis of various pharmaceutically relevant heterocyclic analogues and drugs is one of the leading area of interest [45]. It is obvious that this approach for direct access to a myriad category of *N*-heterocyclic compounds will supplement new paradigms to the existing strategies of drug development. In this direction, several groups reported significant medicinal application of novel *N*-heterocyclic compounds [46,47,48,49,50]. However, the environmentally benign synthesis of drug molecules is not very common in practice. The present review aims to emphasize various environmentally benign and straight-forward synthetic approaches for direct access to *N*-heterocyclic scaffolds of biological importance. It is primarily categorized based on the green alternatives used for the synthesis of *N*-heterocyclic compounds along with further subcategorization according to the class of these compounds.

## 2. Greener Access to Bioactive *N*-Heterocyclic Compounds

### 2.1. Microwave-Assisted Synthesis

#### 2.1.1. Pyrazole Derivatives

Pyrazole derivatives possesses significant potency in drug design due to their prominent therapeutic importance. This unique structural feature has led to diverse bioactivities ranging from antihypertensive and antiviral to neuroprotective activity.

**Scheme 1:**
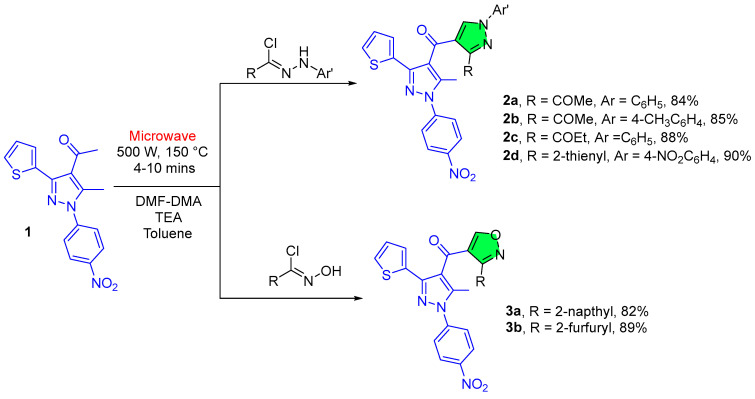

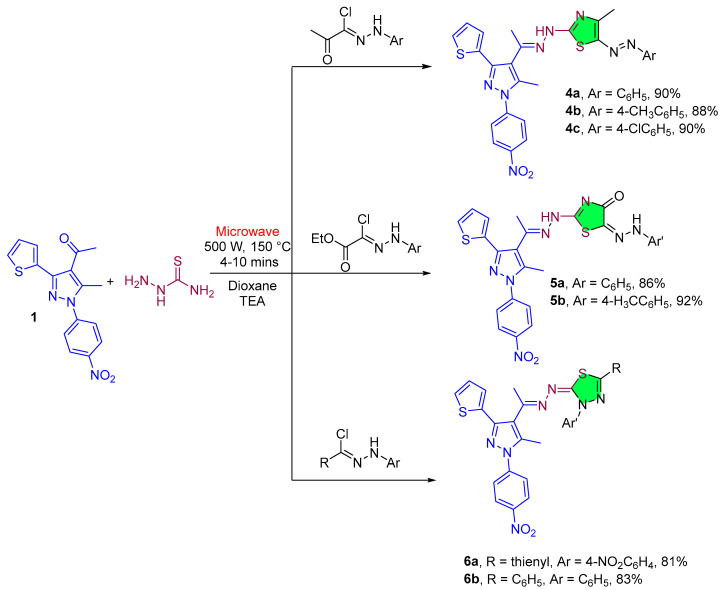
Synthesis of pyrazole derivatives via MCR under microwave heating.

**Figure 3:**
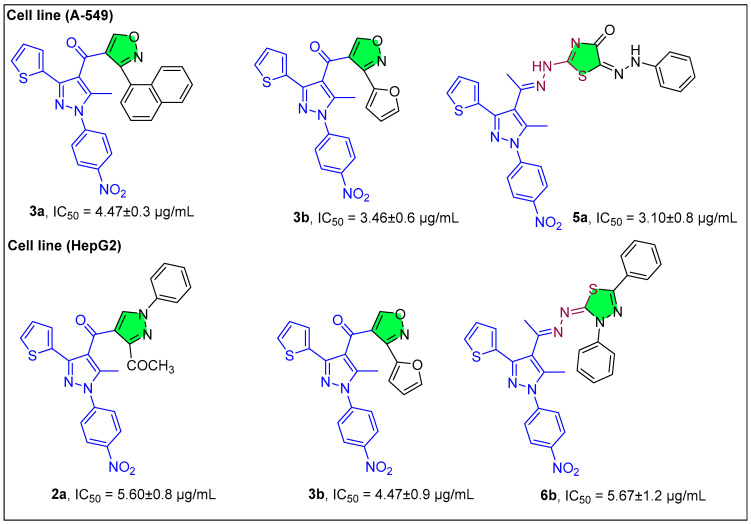
Bioactivity of pyrazole derivatives in cell lines A-549 and HepG2.

In this regard, Gomha and co-workers synthesized various novel pyrazole-based azoles via multi component reaction (MCR) of three substrates under microwave heating for the evaluation of anticarcinogenic effects against hepatocellular carcinoma (HepG2) and A-549 (human lung cancer) cell lines compared to cisplatin as the reference drug in an MTT assay (Scheme 1) [51]. The target pyrazole/oxazole derivatives can be achieved via the 1,3-cycloaddition reaction of enaminone generated from acetylpyrrole and DMF-DMA, to nitrile imines/nitrile oxides, formed in situ from α-ketohydrazonoyl halides or hydroximoyl chloride in the presence of a catalytic amount of TEA. The protocol was further extended by performing a one-pot MCR with acetyl pyrazole and thiosemicarbazide followed by α-keto hydrazonoyl halides in cat TEA/dioxane under MWI to afford the arylazothiazole derivatives. The IC_50_ values of the most potent compounds against A-549 and HepG2 cancer cell lines are shown in Figure 3.

#### 2.1.2. Tetrazole Derivatives

**Scheme 2:**
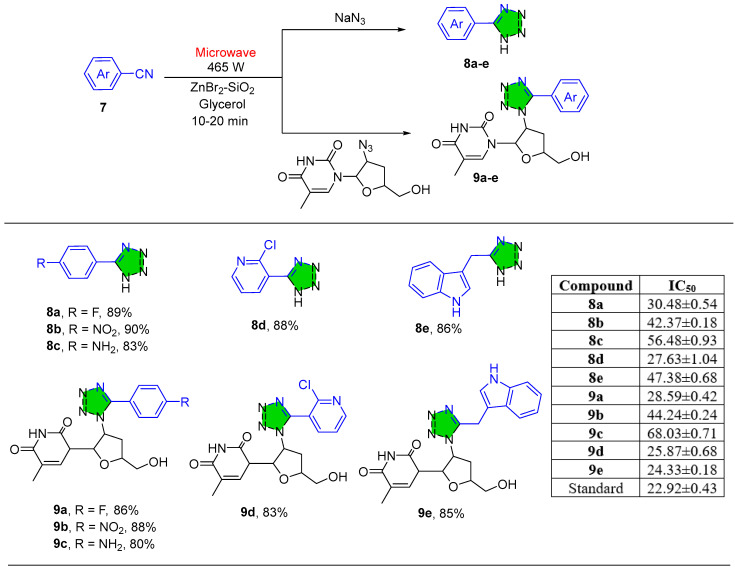
Synthesis of pyrazole derivatives via MCR under microwave heating and their biological evaluation.

Tetrazole derivatives are a precursor of a variety of potential medicinal and drug candidates. Many FDA-approved drugs available in the market possess a tetrazole scaffold as the core structure. Ghamarthi et al. reported a direct access to pharmaceutically active tetrazole via [2+3] cycloaddition reaction of aryl nitriles with sodium azide in the presence of the heterogenous catalyst ZnBr_2_-SiO_2_ and glycerol solvent under microwave irradiation (Scheme 2) [52]. The synthesized compounds show good antioxidant property determined by radical scavenging activity. The existence of radicals was identified through the detection of prominent absorption maximum at 517 nm in the 1,1-diphenyl-2-picrylhydrazyl (DPPH). Butylated hydroxytoluene (BHT) was used as the standard antioxidant for the studies. Among the screened candidates, compounds **8d**, **9d,** and **9e** appear to have promising radical scavenging activity. Molecular docking studies reveal good binding affinity of the synthesized compounds towards reverse transcriptase, aromatase, and aurora.

#### 2.1.3. Benzimidazole Derivatives

**Scheme 3:**
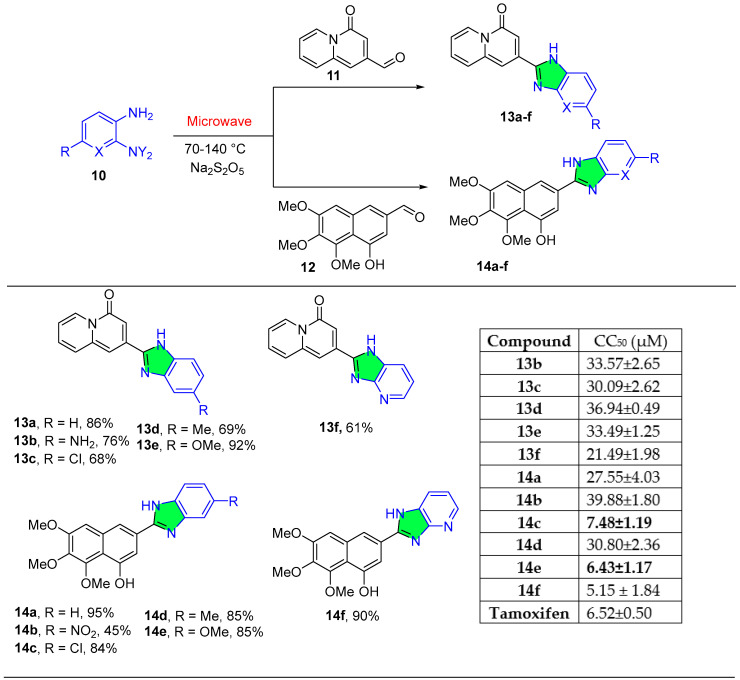
Synthesis and cytotoxic effect of benzimidazole derivatives against MCF-7 cells.

Similar to pyrazole, benzimidazole derivatives are also considered as potent bioactive pharmacophores, having a wide range of application in pharmaceuticals. Therefore, scientists devoted colossal effort to expanding the library of various substituted benzimidazole derivatives with greater diversity. In this context, 2-substituted benzimidazoles have been established as anticancer agents, whereas the 5-chloro/carboxyl functionalized version of 2-substituted benzimidazole displays antitumor activity. Bui et al. established microwave-assisted synthesis of novel benzimidazole derivatives in good-to-excellent yield via the condensation of substituted *o*-phenyldiamine or *o*-nitroaniline with 4-oxo-4H-quinolizinecarbaldehyde (**11**) or naphthalenecarbaldehyde (**12**) in a short period of time (Scheme 3) [53]. Sodium metabisulfite proved to be an ideal oxidant under the reaction condition. The synthesized derivatives were evaluated for their cytotoxic activity against human breast cancer cell line (MCF-7) using tamoxifen as the standard. The product series displayed moderate activity against MCF-7 where the activity increased with the increasing size of the substituents at the C-5 position of benzimidazole ring. However, the exceptional activity with NH_2_ substituent, in spite of the small size, could be explained by the extent of H-bonding interactions with the target. The activity of the **13a**–**f** series was found to be greater as compared to the **12a**–**f** series. Among them, compound **13c** with electron-withdrawing group Cl and **13f** with electron-donating group -OMe have the highest potency for MCF-7 cells, which can be attributed to their greater size. They possess highest potencies to be taken forward in the development of novel compounds with anti-breast-cancer activity.

**Scheme 4:**
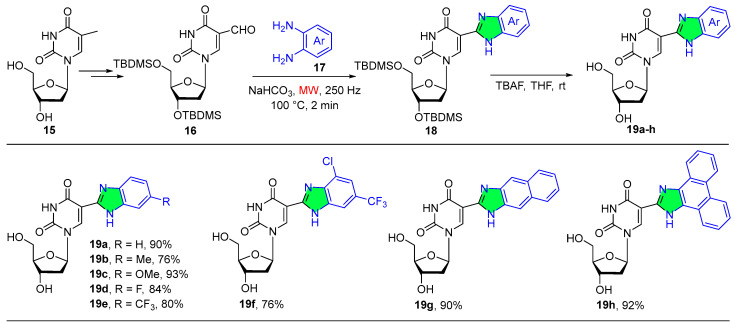
Microwave-irradiated synthesis of sugar–benzimidazole conjugates for antibacterial evaluation.

As evident from the previous report, benzimidazole scaffolds are an important pharmacophore and a potent candidate for the designing of pharmaceutically active molecules. In this direction, a series of new C-5 benzimidazolyl-20-deoxyuridines were synthesized by Engels and coworkers, under solvent-free conditions and microwave irradiation (Scheme 4) [54]. The reaction between 5-formyl-20-deoxyuridine and arylenediamine derivatives using catalytic amounts of NaHSO_3_ generated quantitative yield of the products. All compounds were screened against a series of Gram-positive and Gram-negative bacteria for their antibacterial property. The trifluoromethyl-substituted benzimidazole derivatives show considerable antibacterial activity. The compounds **19a–h** show activity in terms of higher MICs greater than 64 μg mL^−1^ compared to compounds **19d** and **19e,** within which **19e** especially exhibits better antibacterial activity against Gram-positive bacteria *S. aureus* (2 μg mL^−1^), *E. faecalis* (2 μg mL^−1^), *E. faecium* (1 μg mL^−1^), and *S. pneumoniae* (4–16 μg mL^−1^), as compared to the reference drugs ciprofloxacin and linezolid. Additionally, these derivatives displayed exceptional fluorescence activities in the 400–500 nm region.

**Scheme 5:**
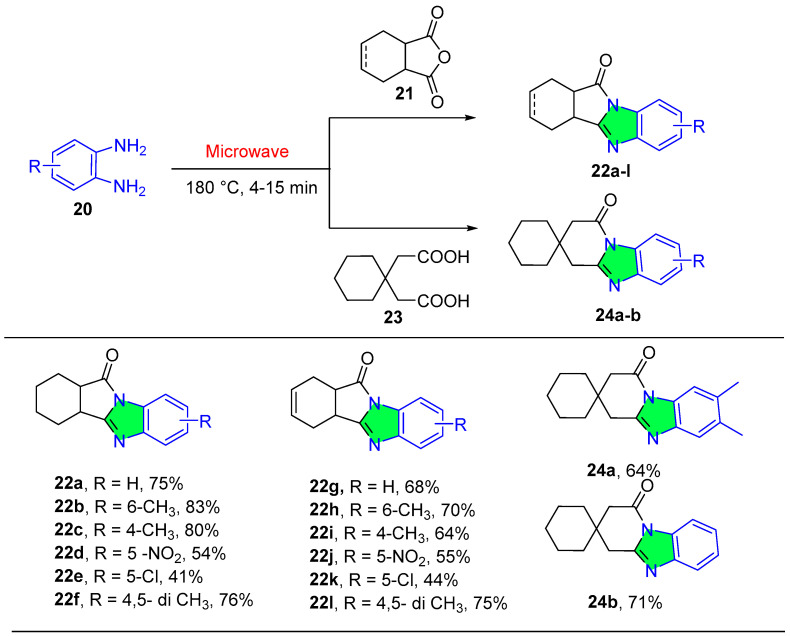
Microwave-assisted synthesis of tetracyclic benzimidazole derivatives.

**Figure 4:**
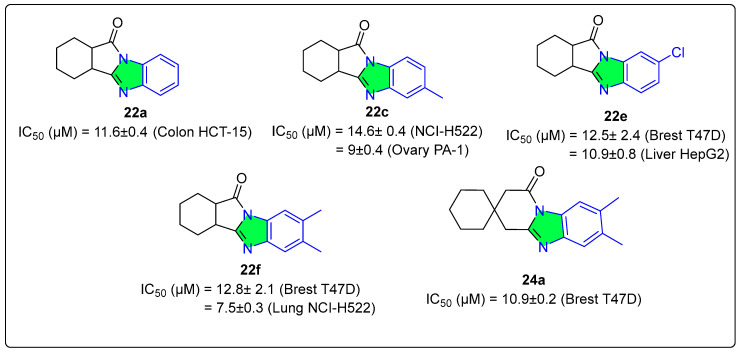
Bioevaluation of benzimidazole derivatives in various cell lines.

Benzimidazoles and perimidines are basic structural entities for the development of new pharmaceutically active molecules and have great significance for medicinal purpose because of their promising biological activities. A series of tetra and pentacyclic benzimidazole and perimidines were generated by A. Sharma and coworkers through greener synthetic approach using microwave irradiation (Scheme 5) [55]. The condensation of various aromatic diamines (**20**) with tetra-/hexahydroisobenzofuran-1,3-dione (**21**) or diacetic acid (**23**) derivatives under microwave irradiation at high temperature for a maximum of 15 min furnished target compounds in moderate-to-good yields. In most of the cases, two regioisomers can be generated during the formation of products. For example, in **22b**, the regioselective formation of one isomer over the other can be attributed to the enhanced reaction of the more nucleophilic amino group ortho to –CH_3_, to form *N*-substituted cyclic imides as the intermediate, which proceeds with further annulation to provide a single regioisomer. All the synthesized derivatives were screened through in vitro analysis for anticancer activity in five different cancer cell lines. Compounds **22a**, **22c**, **22e, 22f**, and **24a** express good antiproliferative activity. Compounds **22a** (colon HCT-15), **22c** (lung NCl H-522, ovary PA-1), **22e** (breast T47D, liver HepG2), **22f** (breast T47D, lung NCl H-522), and **24a** (breast T47D) exhibit moderate-to-good anticancer activity (Figure 4).

**Scheme 6:**
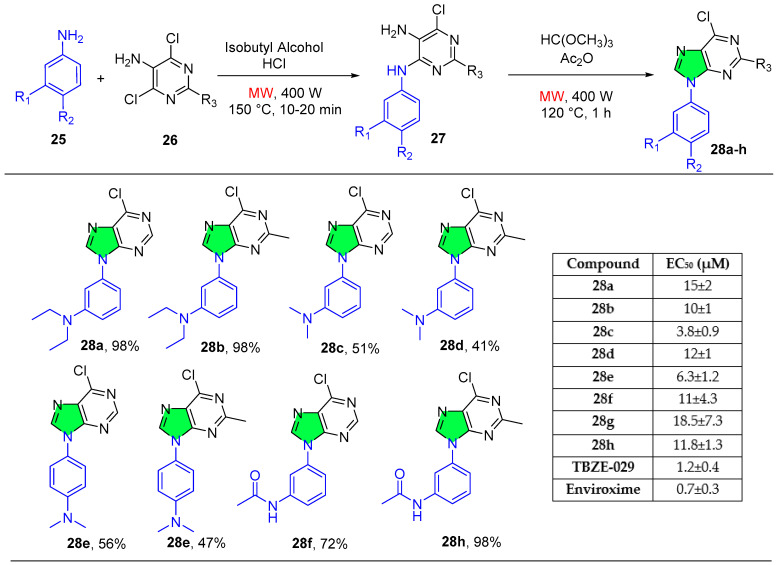
Microwave-assisted synthesis of benzimidazole derivatives and their antiviral activity.

Perpz et al. synthesized 9-aryl-6-chloropurines microwave-assisted two-step protocol involving the reaction of aniline with 4,6-dichloro-5-aminopyrimidines, followed by cyclization with excellent yield. The first step was carried out in isobutanol at 150 °C for 1 h and in the second step, the synthesized compound reacted with trimethylformate in acetic anhydride at 120 °C for 1 h, leading to target molecules (Scheme 6) [56]. The compounds were evaluated for selective antiviral activity on the replication of Coxsackie virus type B3 (CVB3), Nancy strain, in Vero cells. Compounds **28c** and **28g** show efficient activity against the replication Coxackie virus type B4.

#### 2.1.4. Pyrimidine Derivatives

**Scheme 7:**
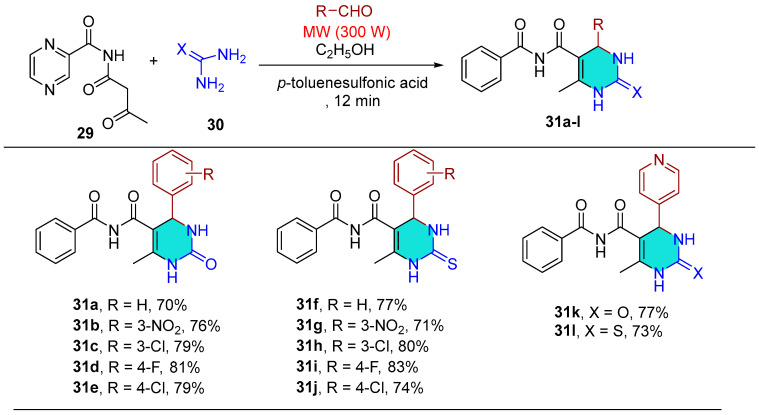
Microwave-assisted synthesis of 1,2,3,4-tetrhydropyrimidines derivatives.

Pyrimidine derivatives are an important class of *N*-heterocyclic compounds due to their therapeutic and pharmaceutical significance. Elumalai et al. accentuated the direct access to novel 1,2,3,4-tetrahydropyrimidine-based derivatives in a 70–83% yield through Biginelli condensation of *N*-(3-oxobutanoyl)pyrazine-2-carboxamide with thiourea/urea and suitable aldehyde in ethanol under microwave irradiation using *p*-toluenesulfonic acid as a catalyst (Scheme 7) [57]. The synthesized tetrahydropyrimidine derivatives show good acetyl and butyl (AChE and BuChE) inhibitor activity (Table 1). It has been identified that AChE promotes facile hydrolysis of acetyl choline to choline and acetate in the process of nerve impulse transmission at cholinergic synapses. Therefore, the inhibition of AChE contributes significantly to the treatment of several neurodegenerative diseases. Inhibition of acetyl and butyl cholinesterase using these pyrimidine derivatives, analyzed by Ellman’s method, showed significant inhibitory activities (IC_50_) (Table 1). Donepezil HCl was used as reference standard in anti-cholinesterase activity. 

The structure activity relationship study reveals that the aryl/heteroaryl substitution at the fourth position of tetrahydropyrimidines is the major entity responsible for acetyl and butyl cholinesterase inhibitor activity. Furthermore, electron-withdrawing groups such as fluoro and chloro in the para-position of 4-aryl substitution decrease the electron density in the ring as a result of inductive effect, leading to an increase in the inhibitor activity. Among the screened analogues, 4-pyridyl substitution with 2-substituted sulphur in tetrahydropyrimidine displays the highest potency, even more than the reference standard. The *N*-(3-oxobutanoyl)pyrazine-2-carboxamide counterpart in the fifth position of tetrahydropyrimidines also contributes to cholinesterase inhibition. 

**Scheme 8:**
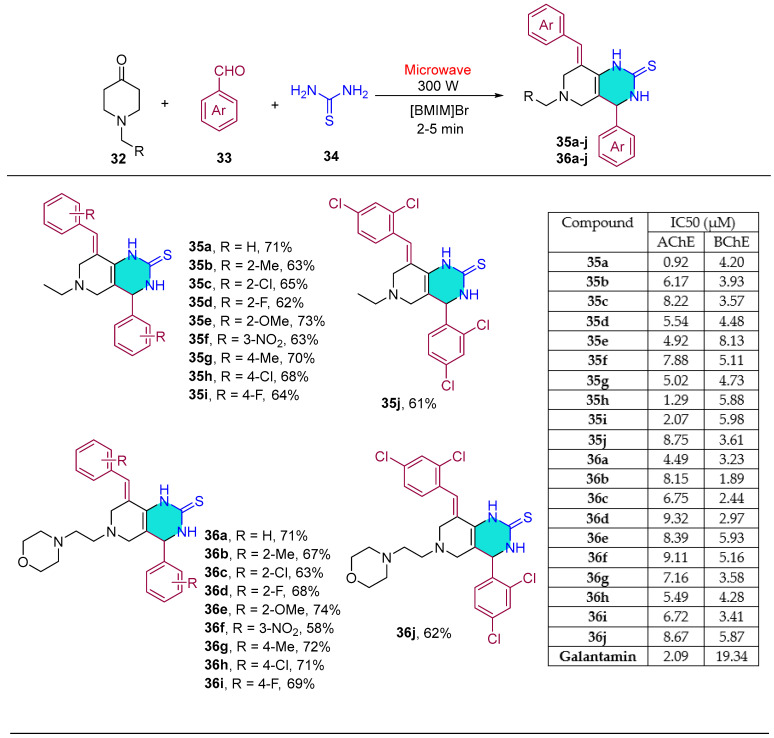
Synthesis of pyridopyrimidine-2-thiones in ionic liquid under microwave conditions for AChE and BChE inhibition.

Multicomponent reactions contribute significantly to the greener approach along with the direct access to pharmaceutically active molecules towards drug development. In this direction, Kumar et al. reported multicomponent synthesis of novel pyridopyrimidine-2-thiones mediated by ionic liquid as green solvents (Scheme 8) [58]. The synthesized derivative shows excellent inhibition against AChE and BChE enzymes with IC_50_ values from 0.92 to 9.11 μM (Scheme 8). The binding site of these inhibitors with their respective active site targets of the enzymes were shown by the molecular modelling. Compared to conventional heating, the reaction carried out with 1 M equivalent of [BMIM]Br under microwave heating produced the target compounds selectively in a shorter time period and with good yields. It was observed that para-substituted derivative possessed greater AChE inhibitory activities over ortho analogues, whereas electronegative groups at para positions displayed preferentially higher binding affinities with AChE enzymes compared to electron-donating substituents. In a comparison of *N*-ethylmorpholine-substituted derivatives in series **36**, *N*-ethyl-substituted derivative in the **35** series shows greater AChE inhibition. However, unsubstituted phenyl derivative **35a** shows the highest AChE inhibitor activity whereas *o*-Me-substituted derivative **36b** shows the highest BChE inhibitor activity. In contrast to the inhibitory activity profile with AChE enzymes, improved BChE inhibition was observed with the electron-donating group compared to electron-withdrawing group. Furthermore, the *ortho*-substituted phenyl analogues of the **35** and **36** series exhibited greater higher BChE inhibitor activity than para-substituted phenyl compounds. Analysis of the structure for pyridopyrimidine-2-thiones was performed through NMR as well X-ray crystallography of **36b,** through which the stereochemistry was also assigned.

**Scheme 9:**
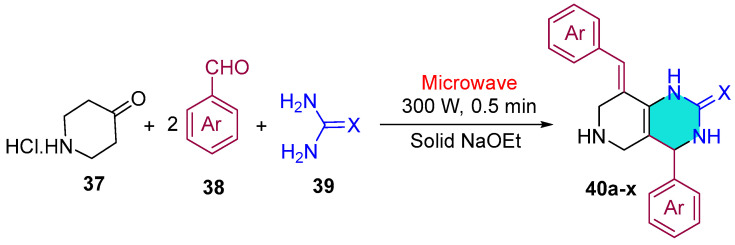
Microwave-assisted synthesis of pyridopyrimidine derivatives.

The microwave-assisted reactions under solvent-free conditions portray the greener aspect of the synthetic protocol significantly. In this direction, V. Murugaiyah and R. S. Kumar synthesized novel pyrido-pyrimidine-2-ones/thiones under eco-friendly, solvent-free, microwave-irradiated reaction conditions in the presence of solid sodium ethoxide via a multicomponent reaction using substituted phenyl aldehyde, urea/thiourea, and unsaturated ketones in excellent yields (Scheme 9) [59]. The in vitro analysis of these products for AChE and BChE inhibition activity shows good result (Table 2). The molecular dynamic stimulation of the pyrido-pyridine derivatives through the 3D structure of specific AChE and BChE enzymes reveals their binding interaction in the active site of the receptors. The inhibition activity against the two cholinesterase enzymes is shown in the table. It is found that the 2-methoxy (**40q**) phenyl group substitution shows the highest potency for AChE and BChE inhibition. However, 1-napthyl (**40x**) substitution also displays good AChE inhibitory activity. It is observed that among C=O and C=S moieties, more polarizable sulphur-containing compounds shows greater inhibition against cholinesterase enzymes. *Ortho*-substituted phenyl derivative shows better activity than the other substituents against BChE enzymes. It was hypothesized that the planar structure in pyridopyrimidine was mandatory for their binding into the active site. Additionally, the greater aromatic residues promote the lodging of the inhibitor into the active binding pocket via hydrophobic interactions with aromatic counterparts, which, in turn, establishes their inhibitory activity, owing to their significant potency as a cholinesterase inhibitor.

**Scheme 10:**
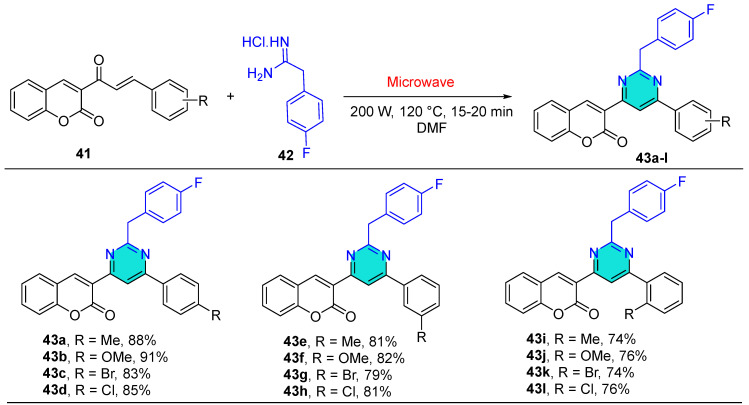
Microwave-irradiated synthesis of coumarin–pyrimidine conjugates.

Fluorine-containing *N*-heterocyclic pharmacophores have contributed to drug discovery significantly, owing to their improvised bioavailability, incremented binding interactions, alteration of the pharmacokinetic and pharmacodynamics properties, and various other aspects. In this context, Hosamani et al. reported a generalized effective and hasty microwave-irradiated synthesis of novel fluorinated coumarin–pyrimidine conjugate as a potent anticancer agent [60]. The condensation of chalconated coumarin with 2-(4-fluorophenyl) acetamidine hydrochloride in DMF solvent at 120 °C led to the additional product in a 74–91% yield (Scheme 10). The synthesized compounds were screened against two anticancer cell lines, A-549 (human lung carcinoma) and MDA-MB-231 (human adenocarcinoma mammary gland) by MTT assay using clinically recommended cisplatin as standard (Table 3). The *p*-chloro phenyl-substituted pyrimidine–coumarin hybrid (**43b**) is found to be the most potent among all others, possessing an IC50 value of 2.15 µM against A-549. In contrast, electron-donating groups such as *p*-methoxy phenyl substitution in **43b** display enhanced potency against MDA-MB-231 cancer cell line. The compounds **43a** and **43b,** possessing the maximum antiproliferative activity against the two cell lines, were identified to cleave DNA fully.

**Scheme 11:**
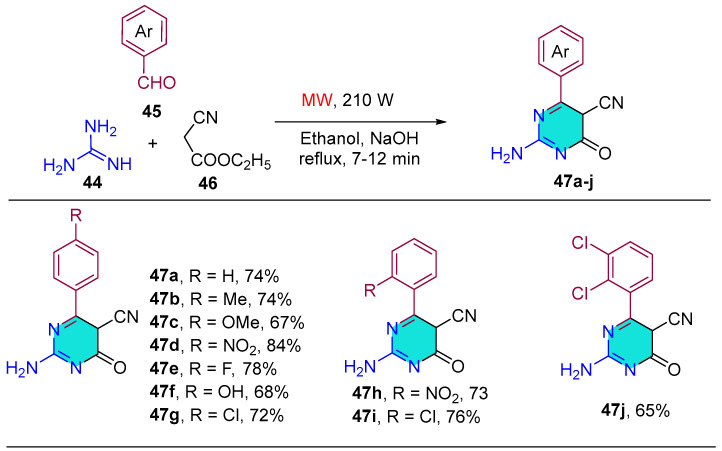
Microwave-assisted synthesis of pyrimidine derivatives.

Panda et al. reported microwave-assisted convenient synthesis of pyrimidine derivatives as potential antitubercular agents. The synthesis was carried out by the condensation of aryl aldehyde, guanidine, and cyanoacetate in the presence of ethanolic NaOH under microwave irradiation for 7–12 min to afford the final compound (Scheme 11) [61]. The synthesized compounds show excellent antitubercular activity against Mycobacterium tuberculosis H37Rv and the clinical isolates, R, S, H, and E-resistant Mycobacterium tuberculosis. Isoniazid was taken as the standard drug. The in vitro antitubercular activity was analyzed by luciferase reporter phage assay method and the antitubercular activity was determined in terms of percentage reduction in the relative light unit (RLU) [Table 4]. The compounds **47d**, **47e**, **47g**, **47h**, **47i**, and **47j** are found to be the most active against at a concentration of 50 μg mL^−1^ and **47e**, **47f**, **47g**, **47i,** and **47j** display antitubercular activity at a concentration of 100 μg mL^−1^. Compounds **47g**, **47h**, and **47j** show excellent activity against the clinical isolates S, H, R, and E resistance of Mycobacterium tuberculosis at a concentration of 50 μg mL^−1^, whereas compounds **47d**, **47f**, **47g**, **47h**, and **47j** show the most potential against resistant strains at a concentration of 100 μg mL^−1^. From the SAR study, it is revealed that the antitubercular activity happens due to the presence of substituents in the aryl ring. The electron-withdrawing groups exhibit promising activity rather than the other derivatives.

#### 2.1.5. Quinoline Derivatives

**Scheme 12:**
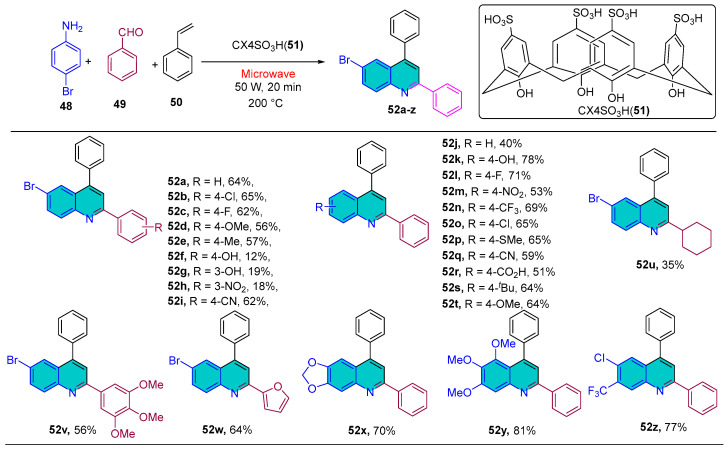
Microwave-irradiated synthesis of quinoline derivatives.

Quinoline is a promising scaffold in pharmaceutically important compounds and frequently exists in clinically tested drug candidates. The development of eco-friendly and efficient strategies for direct access to quinoline analogues have gained escalating interest due to their broad range of biological applications. In this regard, Fernandes and co-workers reported microwave-irradiated synthesis of substituted quinolines in good yields via a three-component condensation reaction of 4-bromoanilene, benzaldehyde and styrene in the presence of 1 mol% *p*-sulfonic acid calixarene (CX4SO_3_H) as a catalyst at 200 °C for 15 min (Scheme 12) [62]. The synthesized compounds were then evaluated for antifungal activity against *C. albicans* (ATCC 10231) and *C. neoformans* (ATCC 32264). The inhibitory activity of quinoline derivative containing 2-furan substitution (**52w**) was analyzed through concentration-dependent studies, which exhibited the highest growth inhibition against *C. albicans* at a concentration of 125 μg/mL. The synthesized quinoline derivatives were evaluated for different cancer cell lines. The quinoline derivative with the 4-methoxyphenyl group showed improved antiproliferative activity against lung cancer. It was concluded that among all others, the significantly active compounds carried 4-fluorophenyl, 3-nitrophenyl, and cyclohexane substituents in a quinoline framework. The presence of activating groups in the phenyl ring enhanced the anticancer activity of the quinoline analogues.

#### 2.1.6. Pyrido-Pyrimidine Derivatives

**Scheme 13:**
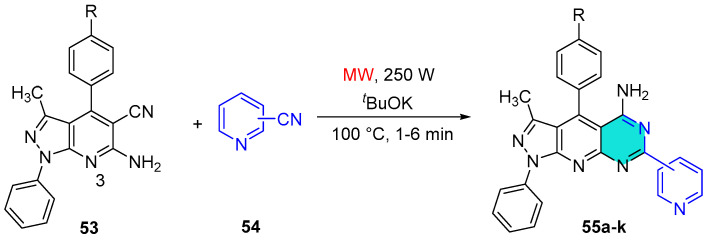
Synthesis of pyrazolo[4,3′:5,6]pyrido[2,3-d]pyrimidine derivatives.

Quiroga and co-workers reported novel fused pyrazolo[4′,3′:5,6]pyrido[2,3-d]pyrimidine derivatives under solvent-free microwave irradiation in the presence of a catalytic amount of solid potassium *tert*-butoxide via a condensation reaction of *o*-aminonitriles and cyanopyrimidines with decent yields (Scheme 13) [63]. The synthesized pyrimidine derivatives were subjected to the evaluation of antifungal activity against *Candida albicans* and *Cryptococcus neoformans* strains (Table 5). A critical structural analysis of various derivatives led to the anticipation that the position of nitrogen in the pyrimidyl moiety has no significant role in modulating the antifungal activity. However, it was observed that the variation in the R group of the phenyl substitution has significant influence in improving the overall activity. The compound **55a,** containing chlorophenyl and Pyridin-4-yl substitution, displayed the highest antifungal activity among all others.

#### 2.1.7. 1,2,3-Triazole-Conjugated Benzodiazepine

**Scheme 14:**
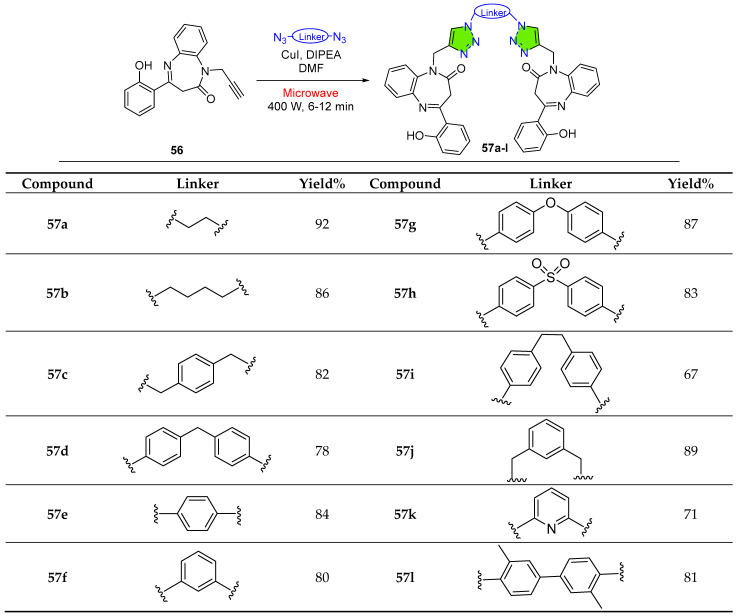
Microwave-assisted click reaction of bisazides with alkynes.

**Figure 5:**
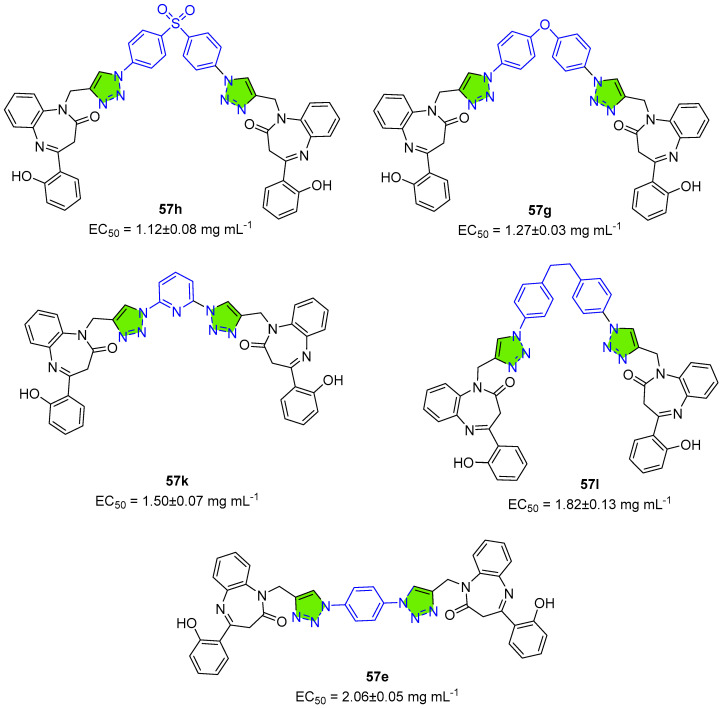
Antimicrobial activities of 1,2,3-triazole-benzodiazepine conjugates.

An array of novel *N*-bis-1,2,3-triazolo-1,5-benzodiazepin-2-ones were established by Msaddek and co-workers through microwave-irradiated click reactions of bisazides with alkyne using Cu(I) as a catalyst and DIPEA as a base in DMF (Scheme 14) [64]. All the synthesized derivatives obtained good yields within 6–12 min, and showed moderate-to-excellent antimicrobial and antioxidant activities (Figure 5). *B. subtilus* (ATCC 6633), *S. epidermis* (CI), *S. aureus* (ATCC 25923), and *S. aureus* (ATCC 29213) are used as Gram-positive bacteria. *E. coli* (ATCC 25922), *K. pneumonia* (CI), *E. fecalis* (ATCC 29212), *S. enterica* (CIP 5262), and *E. faecium* (CI) are used as Gram-negative bacteria. The screening of dimeric 1,5-benzodiazepine-1,2,3-triazole disclosed the compounds **57g**, and **57l** to be active against Gram-positive and **57h** and **57k** against Gram-negative bacteria. Compounds **57h** and **57k** also show higher antifungal activity against *C. glabrata* (ATCC 90030) and *C. keusei* (ATCC 62587) strains. In addition, evaluation of antioxidant activity reveals that compounds **57e, 57g**, **57h**, **57k,** and **57l** show moderate-to-high activity (Figure 5) where Trolox has been taken as the standard reference antioxidant. The bioactivity profile displays the significance of triazole moiety combined with the methylene linker in improvising the potency of benzodiazepine.

**Scheme 15:**
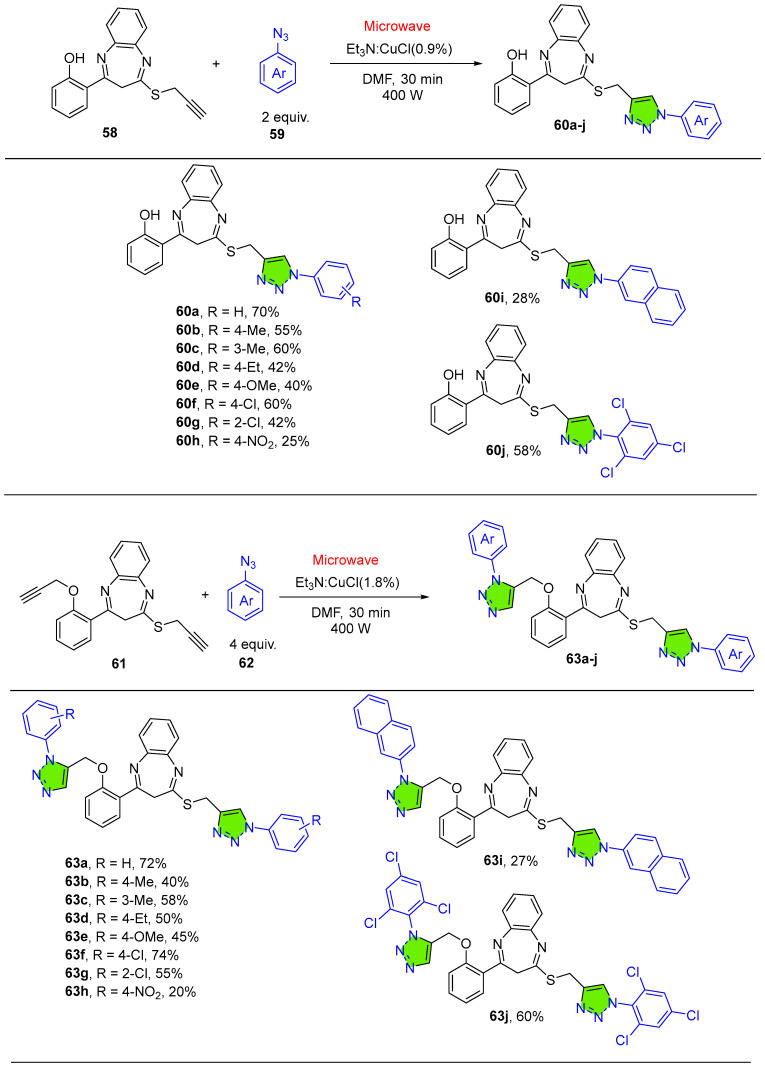
Synthesis of mono and bis-1,2,3-triazole-conjugated benzodiazepines.

Gharbi et al. reported on azide–alkyne cycloaddition reaction under microwave irradiation to synthesize *S*-mono and *S,O*-bis-1,2,3-triazole-linked 1,5-benzodiazepine derivatives (Scheme 15) and evaluated them in vitro for cytotoxic (against MCF-7, HeLa, and A549 cell lines) (Table 6), anti-tyrosinase, and anti-cholinesterase activities (Table 7) [65]. It is observed that among all the derivatives, chlorine-substituted mono 1,2,3-triazolo-benzodiazepine conjugates (**60f**, **60g**, and **60j**) show prominent anti-cholinesterase activity and *p*-chlorophenyl-substituted derivative **60f** expresses the best anti-tyrosinase activity.

#### 2.1.8. Tetrazole-Conjugated Benzodiazepines

**Scheme 16:**
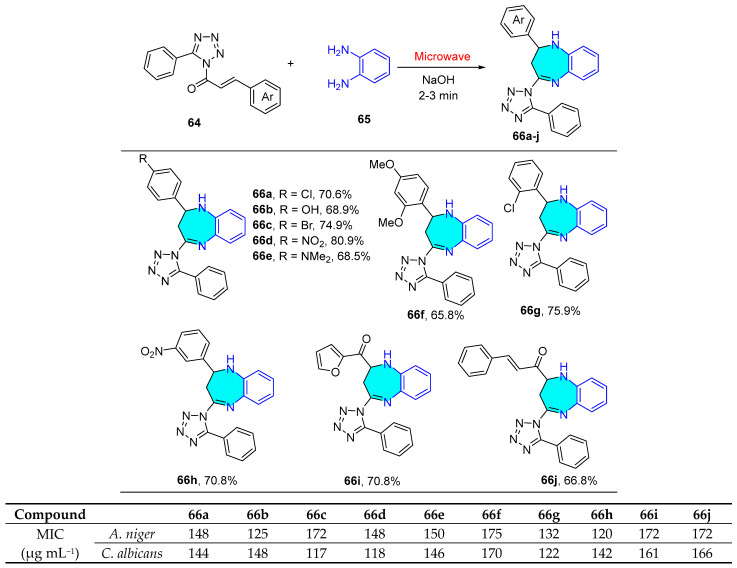
Microwave-assisted synthesis of tetrazole–benzodiazepines conjugates and their antifungal activity.

Bhoge et al. synthesized novel tetrazole-containing benzodiazepines by a cycloaddition reaction of *o*-phenyl diamine and substituted chalcone derivatives under microwave irradiation in the presence of sodium hydroxide under solvent-free conditions (Scheme 16) [66]. The synthesized compounds show moderate-to-good antifungal activity. The tabulated MIC values reflect the antifungal activity of these derivatives against *A. niger* and *C. albicans*. Compounds **66b** and **66h,** containing *p*-OH and *m*-NO_2_-substituted phenyl attachment in the benzodiazepines, show better anti-fungal activity against *A. niger*. Compounds **66c**, **66d**, and **66g**, having *p*-Br, *p*-NO_2,_ and *o*-Cl-phenyl analogues, display good activity against *C. albicans* compared to the others. The activities were comparable with control standard fluconazole, which shows potent activity at MIC of 85 and 110 μg mL^−1^.

**Scheme 17:**
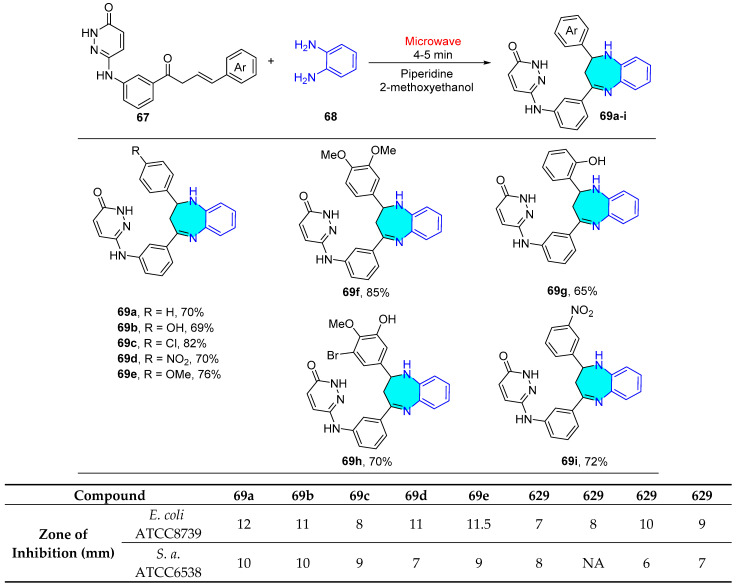
Microwave-irradiated synthesis of 1,5-benzodiazepine derivatives and their antibacterial evaluation.

Pawar and Tupare reported a similar synthesis of 1,5-benzodiazepine analogues as that of Bhoge and coworkers, described in a previous scheme. The green and efficient microwave-irradiated synthesis of 1,5-benzodiazepine derivatives via cycloaddition reaction of *o*-phenyl diamine and substituted chalcone in the presence of a few drops of piperidine and 2-methoxyethanol furnished products in high yields (Scheme 17) [67]. The synthesized compounds show antimicrobial activity against *E. coli* and *S. a.* bacteria. The experiment was performed using the paper disc diffusion plate method. The zone of inhibition of the synthesized compounds are shown in the table in Scheme 17.

**Scheme 18:**
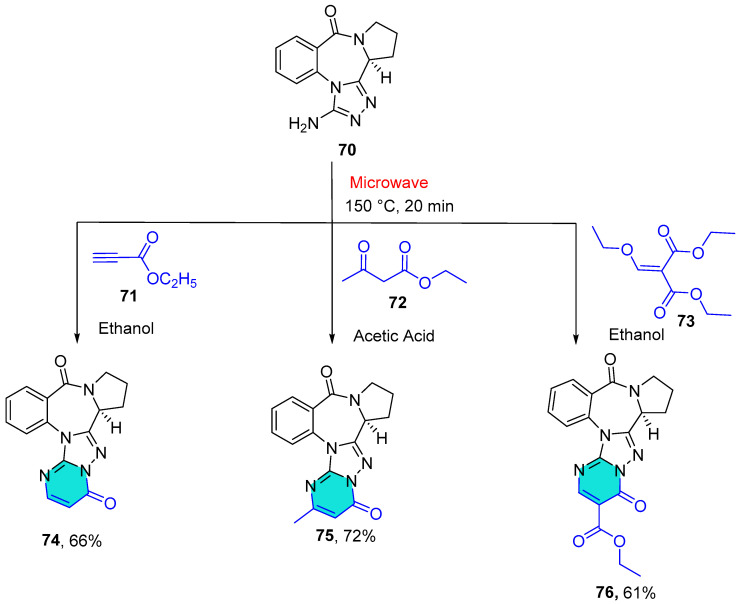
Synthesis of pentacyclic benzodiazepines under microwave irradiation.

Chuang and Wu et al. synthesized novel pentacyclic benzodiazepine derivatives under microwave irradiation via an intramolecular cycloaddition reaction (Scheme 18) [68]. The tricyclic pyrrolobenzodiazepines were important interactive agents with DNA. The tricyclic derivative reacts with ethylpropiolate in ethanol to form pentacyclic derivative **74** with a 66% yield. With ethyl acetoacetate or diethyl ethoxymethylenemalonate in acetic acid solution, it forms compounds **75** and **76,** respectively, under microwave irradiation at 150 °C with good yields. The synthesized derivatives were, therefore, screened for anticonvulsant activities in picrotoxin- and strychnine-induced convulsion models in mice (Table 8). The duration of sleeping time induced by pentobarbital (marked as *) and diazepam induced at 1 mg kg^−1^ shows a significant decrement in the onset of sleep and an increase in the sleeping time. Compound **74** exhibits better sedative property, as interpreted from the in vitro experiment.

### 2.2. Solvent-Free Synthesis

**Scheme 19:**
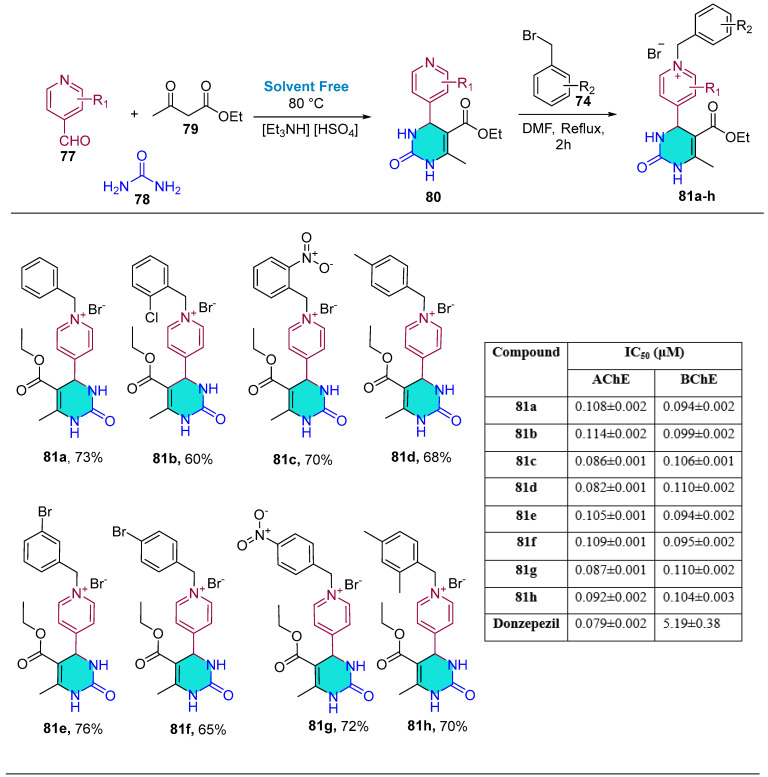
Synthesis of tetrahydropyrimidine-4-yl pyrimidines and their inhibitory activities of cholinesterase enzymes.

Among the variety of heterocycles, tetrahydropyrimidines are versatile building blocks in synthetic organic chemistry and also have major applications in the medicinal and biological fields due to their diverse biopharmaceutical activities. Lotfi and co-workers designed the synthesis of novel tetrahydropyrimidine-4-yl pyrimidine derivatives under solvent-free conditions for the inhibition of cholinesterase enzymes (Scheme 19) [69]. The ionic liquid [Et_3_NH] [HSO_4_] was introduced in a catalytic amount to facilitate the reaction. The synthesized tetrahydropyrimidines are found to have significant inhibitory activities against BChE and are more potent than donepezil, taken as the standard. They also display good AChE inhibition activities, exhibiting IC_50_ values within 0.08 to 0.1 µM. It was realized that 4-methyl-substituted derivatives with an IC_50_ value of 0.082 µM have the greatest potency among all others. The compounds **81c** and **81g**, bearing *o*- and *p*-NO_2_ groups in the phenyl substituent, display considerable anti-AChE activity. However, altering the electron-withdrawing –NO_2_ substitution with the electron-donating -Me group in the para position of the phenyl ring led to the most potent compound **81d**, exhibiting the maximum inhibitory activity against AChE enzymes (IC_50_ = 0.082 µM). It is noteworthy to mention that the same compounds with -Me and –NO_2_ substitutions show comparatively weaker inhibitory activities against BChE.

**Scheme 20:**
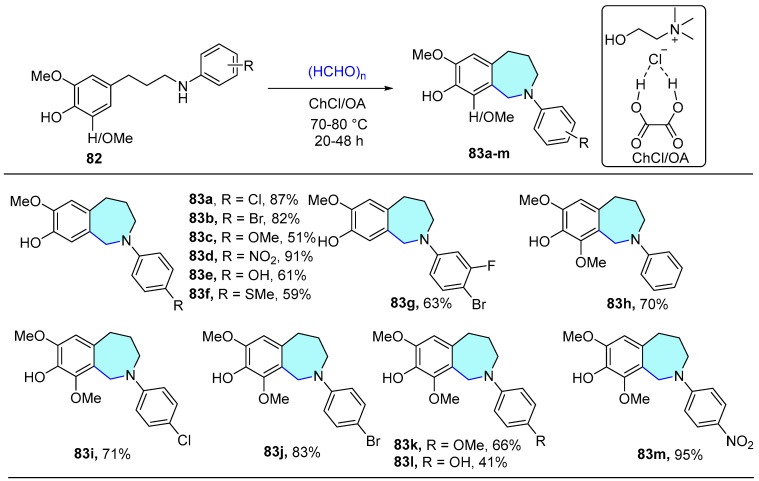
Solvent-free synthesis of substituted 3,4-dimethoxy-phenyl ethylamines.

Renewable feedstocks are an attractive sources for platform and value-added chemicals that function as suitable substrates for direct access to pharmaceutically relevant compounds. In this context, the aromatic analogues derived from the depolymerization of lignin can lead to benzazepines in two to three steps, limiting the generation of waste. Benzazepine derivatives are an important class of scaffolds prioritized in the pharmaceutical industry. In this direction, substituted 3,4-dimethoxy-phenyl ethylamine derivatives were subjected to react with choline chloride (ChCl) and oxalic acid (OA) by Barta et al. under solvent-free reaction conditions to produce substituted benzazepine derivatives in good yield and selectivity (Scheme 20) [70]. The synthesized derivatives were screened for inhibitory activity against *Escherichia coli K12* and *Staphylococcus aureus*. 

## 3. Heterogeneous Catalysis

**Scheme 21:**
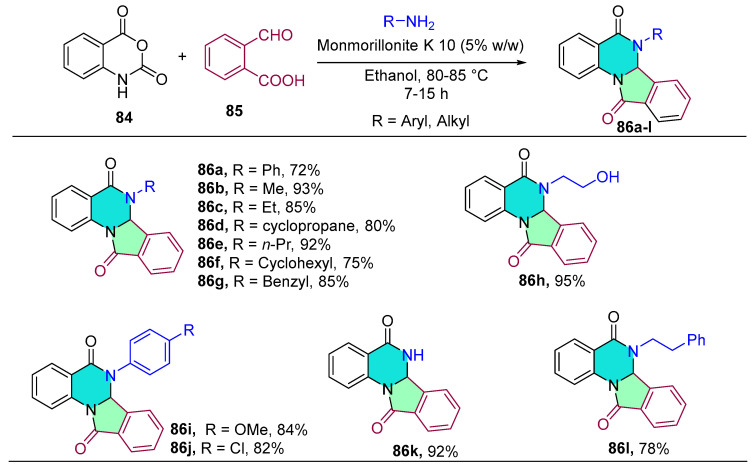
Synthesis of isoindolo[2,1-a]quinazolines as potent inhibitors of TNF-α.

Heterogeneous catalysis has contributed enormously towards the development of green and sustainable strategies toward the synthesis of heterocyclic and carbocyclic entities. Furthermore, various metallic and non-metallic heterogeneous catalysts have been employed for the direct access to medicinally relevant *N*-heterocyclic compounds. In this regard, Manojit et al. reported the application of heterogeneous catalysis in green synthesis of new isoindolo[2,1-*a*]quinazoline derivatives, which act as potent inhibitors related to TNF-α (Scheme 21) [71]. TNF-α is known to be a prime cytokine mediator taking part in the inflammatory response, which can act as a marker in the inflammatory disorder. The reaction of isatoic anhydride, aniline, and 2-formylbenzoic acid in the presence of 5% (*w*/*w*) montemorillonite K10 in ethanol as solvent produced quinazoline derivatives in moderate-to-excellent yields. The synthesized compounds were evaluated for in vitro inhibition of TNF-α. In the series of quinazoline derivatives, compounds **86h**–**k** exhibit considerable inhibitory activity, within which the compound **86k** executes dose-dependent activity with an IC50 of 9.33 µM that could be supported by docking studies. A sturdy interaction with the hydrophobic pockets generated from glycine, leucine, and tyrosine residue might have contributed to the lower binding energy.

**Scheme 22:**
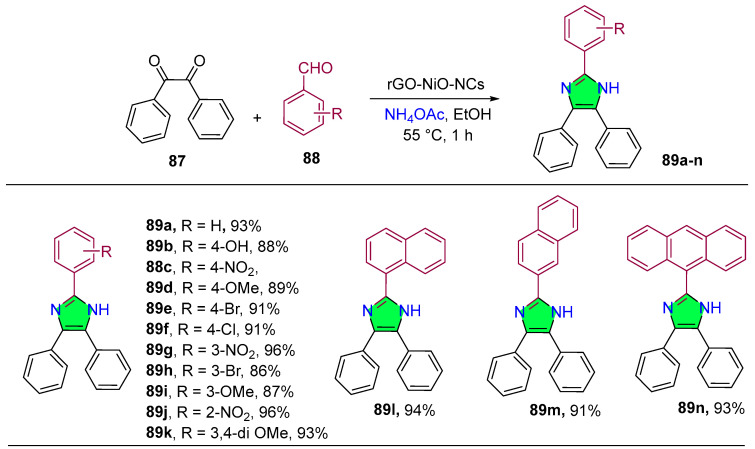
Synthesis of imidazole scaffolds with reduced graphene oxide/NiO nanocomposites.

Imidazole derivatives have several biological applications owing to their significant frequent occurrence in the field of synthetic and natural product chemistry. Masram and co-workers synthesized densely substituted imidazole scaffolds by using reduced graphene oxide/NiO nanocomposites (rGO–NiO-NCs) as economic, environmentally benign, reusable, and efficient nanocatalysts for the in vitro evaluation of DNA-binding inhibition of the imidazole derivatives with ethidium bromide (EB, Scheme 22) [72]. The screening of trisubstituted imidazole derivatives reveals that **89n**–**l** have the enhanced capability to displace EB, which is further validated by molecular docking. Ammonium acetate was used as the main nitrogen source in the construction of imidazoles. The *m*–OMe and *m*–Br functionality in the aryl counterpart decrease the rate of the reaction, producing low yield of products as compared to *ortho*- and *para*-aromatic aldehydes.

**Scheme 23:**
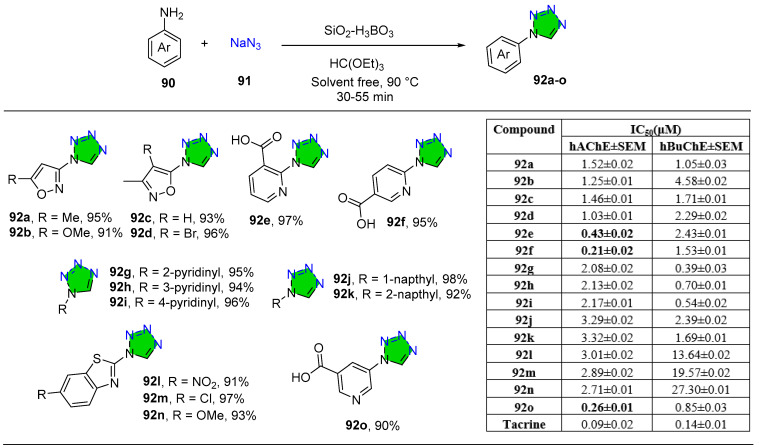
Synthesis of tetrazole derivatives and their bioevaluation against hAChE and hBuChE.

Parveen and co-workers reported solvent-free, green, and sustainable synthesis of tetrazole derivative, which was promoted by the reusable heterogenous catalyst SiO_2_–H_3_BO_3_ (Scheme 23) [73]. This eco-friendly condensation of aryl amine with sodium azide in the presence of triethyl *ortho*-formate produces target tetrazole with an excellent yield. The synthesized tetrazole derivatives were screened for AChE and BuChE inhibition studies. Compounds **92e**, **92f**, and **92o** show the most promising inhibition activity against AChE. Electron-donating groups show greater inhibition against AChE than electron-withdrawing groups. All the synthesized compounds show moderate inhibition against BuChE.

## 4. Ultrasound-Mediated Reactions

**Scheme 24:**
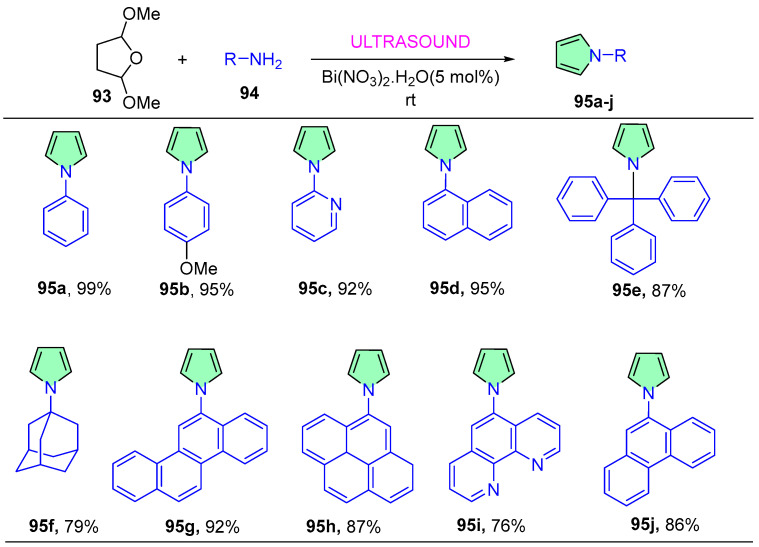
Ultrasound mediated-synthesis of N-substituted pyrrole derivatives.

The ultrasound-mediated synthetic strategies for the direct access to *N*-heterocyclic products exemplify the importance of this approach towards the development of green methodologies. *N*-substituted pyrrole derivatives obtained through ultrasound-promoted reactions plays an important role in this motivating green synthesis. Pyrroles are the basic structural unit in different classes of pharmaceutically important molecules. One of the conventional methods for the synthesis of pyrroles is the Pall–Knorr reaction. Banik and Short synthesized ultrasound-mediated novel *N*-substituted pyrrole derivatives via an eco-friendly and solvent-free route (Scheme 24) [74]. *N*-substituted pyrroles can be achieved by the reaction of 2,5-dimethoxytetrahydrofuran with various amines in the presence of a catalytic amount of bismuth nitrate pentahydrate at room temperature. The novel pyrrole derivatives were subjected to in vitro cancer cell lines such as liver cancer (HepG2 and Hepa 1-6), colon cancer (HT-29 and Caco-2), cervical cancer (HeLa), and NIH3T3 cells to assess their cytotoxicity (Table 9). Compounds **95i** and **95j** show excellent activity against liver cancer cell lines and others. From the cell viability study, compounds **95i** and **95j** efficiently reduced hepa1–6 cell viability at doses 2.5 μM and 5 μM, respectively. However, these two compound are not capable of reducing the viability of normal primary hepatocytes even when used at dosages of 10 μM. Therefore, **95i** and **95j** show distinct inhibitory effects on the viability of cancer cells when compared to normal cells.

**Scheme 25:**
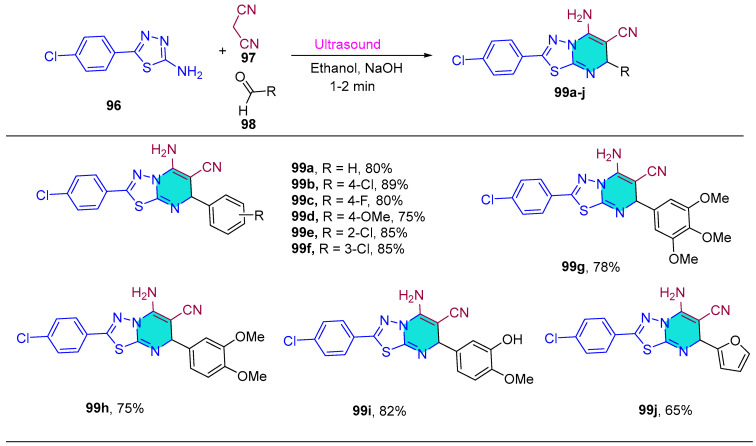
Ultrasound-promoted synthesis of novel pyrimidine derivatives.

Pyrimidine and its derivatives have been recognized as important heterocyclic scaffolds due to their ever-growing chemical and biological significance in various fields. Nikalje et al. reported ultrasound-mediated synthesis of novel pyrimidine derivatives in good-to-excellent yields via a one-pot three-component reaction of 5-(4-chlorophenyl)-1,3,4-thiadiazol-2 amine, aromatic aldehydes, and malononitrile in the presence of a catalytic amount of NaOH (Scheme 25) [75]. The synthesized compounds were then evaluated for anticancer activity against human tumor cell lines using 5-flurouracil as the standard drug. Among the screened analogues, compounds **99d**, **99g**, **99h**, and **99i** were identified as the most potent inhibitors against cell growth (Table 10). From the SAR studies, it was anticipated that the pyrimidine derivatives with electron-donating groups would have relatively higher potency against cancer cell lines as compared to electron-withdrawing analogues. It was also realized that the replacement of the phenyl ring by a furan ring leads to diminution in anticancer activity. A strong binding interaction with the active site of the thymidylate synthase enzyme was demonstrated from docking results, along with the drug-like property of the compounds analyzed from ADME studies.

## 5. Biocatalyst-Mediated Reaction

**Scheme 26:**
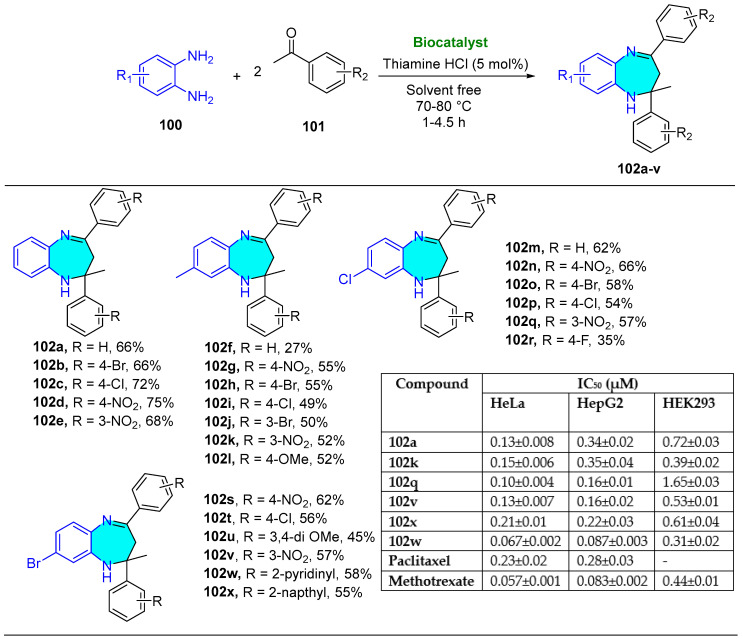
Synthesis of substituted 1,5-benzodiazepines using biocatalyst and their IC_50_ values in different cancer cell lines.

Biocatalysis is an important aspect of green synthesis, which executes selective synthetic transformations in good yields. In this context, *N*-heterocyclic compounds are synthesized efficiently through the introduction of biocatalysts as a green reagent. Desai and co-workers introduced direct access to substituted 1,5-benzodiazepines in good yields using biocatalyst thiamine hydrochloride under solvent-free conditions at 70–80 °C via a condensation reaction with substituted *o*-phenyl diamine and substituted acetophenone (Scheme 26) [76]. The synthesized compounds show good in vitro anticancer activity against HeLa, HEPG2, and HEK-293 via MTT assay. The IC_50_ values vary from 0.067 to 0.35 µM and are found to be superior to paclitaxel and compatible with the methotrexate drug. The derivative **102w** is found to be active against both HeLa and HEPG2. The combined effect of **102w** and methotrexate is highly effective, with IC_50_ values of 0.046 ± 0.002 µM and 0.057 ± 0.002 µM against HeLa and HEPG2 cell lines, respectively. In the case of compound **102w**, the cell viability significantly decreases with the increasing concentration of the compound. The 1,5-benzodiazepines **102d** (IC_50_ = 0.514 ± 0.003 μM), **102k** (IC_50_ = 0.404 ± 0.002 μM), and **102w** (IC_50_ = 0.156 ± 0.003 μM) also display decent tyrosine kinase enzyme inhibition activities as analyzed from the bioinformatics data compared to erlotinib (IC_50_ = 0.18 ± 0.04 μM), which was used as positive control.

## 6. Conclusions

The present collection demonstrates the synthesis of medicinally important *N*-heterocyclic compounds via green and sustainable approaches facilitating drug design. The environmental issues associated with chemical and pharmaceutical industries urges the improvisation of synthetic methodologies towards greener and cleaner versions. The elimination of hazardous side products, in addition to the simple purification steps and utilization of eco-friendly mild reaction conditions, aims to reduce the environmental burden in the near future. The *N*-heterocyclic compounds that constitute a major fraction of FDA-approved drugs are important pharmacophores for drug design and development, demanding a greener synthetic approach. However, the existing synthetic protocols developed for direct access to biologically relevant *N*-heterocyclic compounds often fail to pertain the principle of green chemistry. In this regard, several groups introduced green and sustainable approaches to *N*-heterocyclic scaffolds with or without biological importance. However, the development of eco-friendly methods directly leading to potent bioactive *N*-heterocyclic candidates along with their derivatives are limited.

In this direction, the present review encompasses various synthetic protocols under microwave irradiation, ultrasound, and solvent-free conditions, heterogeneous methods or biocatalysis, etc. However, certain challenges exist in terms of development and generalization of greener protocols to a larger substrate scope. The future aspect of green chemistry in drug development program aims toward the generation of *N*-heterocyclic compounds through more vigorous exploration of various new and efficient sustainable protocols for the generalization of a diverse array of substrates as well as scaling-up for industry applications.

## Figures and Tables

**Table 1:** In-vitro acetyl and butyl cholinesterase inhibitor activity of tetrahydropyrimidine derivatives.

Compound	IC_50_ (μM) ± SEM	
	AChE	BuChE
**31a**	5.35 ± 0.01	7.21 ± 0.01
**31b**	2.54 ± 0.01	5.93 ± 0.01
**31c**	1.21 ± 0.01	4.96 ± 0.01
**31d**	0.86 ± 0.01	4.84 ± 0.01
**31e**	0.94 ± 0.01	4.75 ± 0.01
**31f**	5.26 ± 0.01	6.75 ± 0.01
**31g**	1.82 ± 0.01	5.38 ± 0.01
**31h**	1.05 ± 0.01	4.31 ± 0.01
**31i**	0.75 ± 0.01	3.93 ± 0.01
**31j**	0.88 ± 0.01	4.13 ± 0.01
**31k**	0.19 ± 0.01	3.92 ± 0.01
**31l**	0.11 ± 0.01	3.46 ± 0.01
**Donepezil HCl**	0.13 ± 0.01	3.58 ± 0.01

**Table 2:** In vitro acetyl and butyl cholinesterase inhibition activity of pyridopyrimidine derivatives.

Compound	X	Ar	Yield%	IC_50_ (μΜ)	
				AChE	BChE
**40a**	O	C_6_H_5_	94	68.73	2.86
**40b**	O	2-CH_3_C_6_H_4_	91	40.42	5.22
**40c**	O	2-ClC_6_H_4_	90	24.14	2.51
**40d**	O	2-FC_6_H_4_	89	18.59	3.31
**40e**	O	2-OCH_3_C_6_H_4_	93	40.23	10.70
**40f**	O	3-NO_2_C_6_H_4_	91	28.27	10.34
**40g**	O	4-BrC_6_H_4_	88	15.86	6.22
**40h**	O	4-CH_3_C_6_H_4_	90	46.52	7.09
**40i**	O	4-ClC_6_H_4_	92	36.84	16.73
**40j**	O	4-FC_6_H_4_	94	44.23	18.90
**40k**	O	2,4-Cl_2_C_6_H_4_	91	29.86	23.51
**40l**	O	1-napthyl	92	13.16	5.39
**40m**	S	C_6_H_5_	94	19.27	3.78
**40n**	S	2-CH_3_C_6_H_4_	85	39.72	3.07
**40o**	S	2-ClC_6_H_4_	95	32.72	2.91
**40p**	S	2-FC_6_H_4_	89	40.43	6.50
**40q**	S	2-OCH_3_C_6_H_4_	94	0.80	1.18
**40r**	S	3-NO_2_C_6_H_4_	88	34.31	8.09
**40s**	S	4-BrC_6_H_4_	93	11.88	1.65
**40t**	S	4-CH_3_C_6_H_4_	95	37.47	6.27
**40u**	S	4-ClC_6_H_4_	92	2.25	6.26
**40v**	S	4-FC_6_H_4_	90	37.22	28.82
**40w**	S	2,4-Cl_2_C_6_H_4_	91	26.25	49.2
**40x**	S	1-napthyl	92	1.37	5.58
**Galantamine**	-	-	-	2.09	19.34

**Table 3:** Anticancer activity of pyrimidine–coumarin against cell lines A-549 and MDA-MB-231.

Compound	Cytotoxicity (IC_50_) in μM	
	A-549	MDA-MB-231
**43a**	16.73 ± 1.42	4.16 ± 0.37
**43b**	16.11 ± 1.21	2.23 ± 0.19
**43c**	4.32 ± 0.53	24.43 ± 2.56
**43d**	2.15 ± 0.12	16.53 ± 1.61
**43e**	24.31 ± 2.38	16.42 ± 1.42
**43f**	22.41 ± 2.51	8.42 ± 0.73
**43g**	8.43 ± 0.64	26.79 ± 2.79
**43h**	4.64 ± 0.59	8.31 ± 0.83
**43i**	25.63 ± 2.58	4.62 ± 0.59
**43j**	21.72 ± 2.11	16.16 ± 1.31
**43k**	8.73 ± 0.84	28.57 ± 2.43
**43l**	8.56 ± 0.76	16.74 ± 1.82
**Cis-platin**	1.89 ± 0.09	3.5 ± 0.21

**Table 4:** Antitubercular study of pyrimidine derivatives.

Compound	Reduction in RLU (%)			
	*M. tuberculosis* H37Rv		Clinical Isolate: S, H, R, and E Resistant *M. tuberculosis*	
	50 μg mL^−1^	100 μg mL^−1^	50 μg mL^−1^	100 μg mL^−1^
**47a**	41.62	47.48	43.62	47.76
**47b**	44.46	48.64	44.37	49.83
**47c**	44.85	51.68	38.76	47.24
**47d**	54.76	58.46	43.35	54.85
**47e**	61.45	67.84	40.78	47.43
**47f**	47.65	53.76	48.87	52.66
**47g**	52.67	57.86	54.87	58.48
**47h**	62.47	66.82	51.62	56.64
**47i**	50.36	62.73	38.84	42.77
**47j**	56.84	61.68	56.64	61.46
**Isoniazid**	81.57		84.58	

**Table 5:** Antifungal activity of pyrido-pyrimidine derivatives.

Compound	R	Pyridine Substitution in 48	Yield%	Conc. (μg/mL)	% of Inhibition		Conc. (μg/mL)	% of Inhibition	
					*C. neoformans*	*C. albicans*		*C. neoformans*	*C. albicans*
**55a**	Cl	Pyridin-4-yl	61	250	91.7 ± 2.8	78.3 ± 0.3	125	57.3 ± 0.7	31.0 ± 1.9
**55b**	Cl	Pyridin-3-yl	57	250	17.5 ± 2.7	1.85 ± 0.1	125	16.9 ± 1.8	0
**55c**	Cl	Pyridin-2-yl	42	250	81.6 ± 1.9	8.12 ± 0.7	125	148.6 ± 0.38	4.3 ± 0.4
**55d**	OMe	Pyridin-4-yl	60	250	29.9 ± 1.9	7.3 ± 1.2	125	19.7 ± 0.6	3.0 ± 0.6
**55e**	OMe	Pyridin-3-yl	61	250	72.5 ± 0.9	62.2 ± 2.3	125	20.7 ± 0.4	27.12 ± 1.1
**55f**	OMe	Pyridin-2-yl	42	250	79.7 ± 1.8	63.8 ± 2.0	125	51.3 ± 1.5	23.7 ± 1.8
**55g**	Me	Pyridin-4-yl	62	250	57.3 ± 1.1	76.9 ± 1.3	125	33.09 ± 0.3	25.4 ± 1.5
**55h**	Me	Pyridin-3-yl	56	250	24.7 ± 1.4	58.5 ± 1.3	125	7.9 ± 2.1	31.2 ± 1.8
**55i**	Me	Pyridin-2-yl	48	250	64.5 ± 2.9	11.0 ± 0.6	125	44.6 ± 1.6	3.2 ± 0.9
**55j**	3,4-OCH_2_O	Pyridin-4-yl	59	250	12.9 ± 1.1	22.1 ± 1.5	125	10.5 ± 0.9	11.3 ± 0.9
**55k**	3,4-OCH_2_O	Pyridin-3-yl	50	250	64.5 ± 1.5	50.2 ± 1.7	125	62.5 ± 1.0	34.9 ± 1.7
**Amphotericin B**	-	-	-	250	100	100	125	100	100

**Table 6:** Cytotoxic activity of synthesized compounds in terms of IC_50_ values in different cancer cell lines.

Compounds		IC_50_ (μM)	
	MCF-7	HeLa	A549
**60a**	42.0 ± 2.0	47.0 ± 3.0	>100
**60b**	55.0 ± 1.0	>100	>100
**60c**	70.0 ± 2.0	>100	>100
**60d**	59.0 ± 2.0	>100	>100
**60e**	45.0 ± 1.0	>100	>100
**60f**	18.0 ± 1.0	25.0 ± 2.0	38.0 ± 3.0
**60g**	29.0 ± 2.0	50.0 ± 2.0	32.0 ± 2.0
**60h**	18.0 ± 2.0	13.0 ± 1.0	>100
**60i**	>100	>100	>100
**60j**	15.0 ± 1.0	40.0 ± 2.0	58.0 ± 2.0
**63a**	33.0 ± 2.0	62.0 ± 2.0	>100
**63b**	40.0 ± 2.0	>100	>100
**63c**	51.0 ± 3.0	>100	>100
**63d**	42.0 ± 2.0	>100	>100
**63e**	30.0 ± 2.0	>100	>100
**63f**	16.0 ± 2.0	35.0 ± 2.0	21.0 ± 2.0
**63g**	23.0 ± 1.0	57.0 ± 3.0	17.0 ± 1.0
**63h**	15.0 ± 1.0	22.0 ± 1.0	>100
**63j**	11.0 ± 1.0	52.0 ± 3.0	36.0 ± 2.0
**Doxorubicin**	0.38 ± 0.03	0.36 ± 0.03	-
**Ellipticine**	-	-	0.31 ± 0.04

**Table 7:** IC_50_ values of synthesized compounds against tyrosinase and cholinesterase.

Compound	IC_50_ (μM)	
	Tyrosinase	Cholinesterase
**60a**	146.0 ± 1.0	
**60b**	101.0 ± 2.0	
**60f**	10.0 ± 0.2	29.0 ± 1.0
**60g**		49.0 ± 0.5
**60j**		19.0 ± 0.3
**63a**	181.0 ± 1.0	
**63e**	152.0 ± 1.7	
**63f**	85.0 ± 1.0	105.0 ± 1.0
**63g**	103.0 ± 0.9	152.0 ± 2.0
**63j**		95.0 ± 1.0
**Hydroquinone**	27.0 ± 0.2	^−^
**Galantamine**	-	0.38 × 10^−3^ ± 0.002 × 10^−3^

**Table 8:** Evaluation of anticonvulsant activities on picrotoxin- and strychnine-induced convulsion models in mice.

Compound	Dose (mg/kg)	Picrotoxin		Strychnine	
		Latency (s)	Duration (s)	Latency (s)	Duration (s)
**Vehicle**	-	294.4 ± 26.6	182.9 ± 14.3	304.6 ± 12.4	184.7 ± 14.9
**74**	1	297.5 ± 3.2	181.1 ± 36.3	357.5 ± 30.5	325.5 ± 4.6 ***
**75**	1	312.8 ± 11.2	395.8 ± 38.9 ***	304.6 ± 21.7	171.6 ± 6.1
**76**	1	313.0 ± 18.7	296.9 ± 35.6 *	313.0 ± 18.7	185.6 ± 2.0
**Diazepam**	1	440.5 ± 20.1 **	481.1 ± 18.7 ***	367.1 ± 31.0 *	459.7 ± 24.0 ***

n = 4 mice, * *p* < 0.05, ** *p* < 0.01, *** *p* < 0.001, compared with the vehicle group.

**Table 9:** In vitro evaluation against liver, colon, and cervical cancer cell lines.

Compound	IC_50_ (μM)					
	HepG2	Hepa1–6	Caco-2	HT-29	HeLa	NIG3T3
**95f**	38.6 ± 11.5	19.9 ± 6.1	>50	11.9 ± 1.0	12.9 ± 5.9	24.0 ± 18.5
**95g**	>50	0.7 ± 0.8	>50	>50	>50	>50
**95h**	>50	10.7 ± 0.4	>50	24.3 ± 0.7	17.7 ± 9.9	ND
**95i**	**3.0 ± 1.6**	**3.4 ± 0.4**	ND	**4.2 ± 0.5**	27.9 ± 20.7	**1.9 ± 1.5**
**95j**	13.4 ± 4.8	**3.9 ± 0.3**	>50	>50	>50	**2.1 ± 1.3**
**Cis platin**	7.0	4.0	10.8	16.8	11.7	8.5

**Table 10:** Evaluation of anticancer activity for thiadiazole-fused pyrimidine derivatives.

Compound	GI_50_ μM			
	MCF-7	K-562	HeLa	PC-3
**99a**	38.9	58.3	38.7	34.7
**99b**	88.5	47.9	56.2	38.9
**99c**	80.6	>100	58.1	30.2
**99d**	38.9	54.2	43.8	26.7
**99e**	43.8	57.1	54.3	37.9
**99f**	55.0	60.1	55.7	38.4
**99g**	38.3	58.1	48.6	25.4
**99h**	34.8	54.3	47.9	25.3
**99i**	32.7	55.3	34.3	28.9
**99j**	82.5	>100	60.9	55.3
**5-FU**	32.18	47.03	43.71	12.00

## Data Availability

Data is contained within the article.
